# Global epigenetic immunohistochemical markers reveal developmental connections of distinct cell types during fetal development

**DOI:** 10.1007/s00417-025-07038-7

**Published:** 2026-04-02

**Authors:** Michael T. Jost, Karin U. Loeffler, Frank G. Holz, Amit Sharma, Marie-Therese Schmitz, Annette M. Müller, Martina C. Herwig-Carl

**Affiliations:** 1 Department of Ophthalmology, University Hospital Bonn, Venusberg Campus 1 Gebäude 04/05, 53127 Bonn, Germany; 2 Division of Ophthalmic Pathology, University Hospital Bonn, Bonn, Germany; 3 Institute of Medical Biometry, Informatic and Epidemiology (IMBIE), University Hospital Bonn, Bonn, Germany; 4 Center of Pediatric Pathology and Pathology, University Hospital Cologne, Köln, Germany

**Keywords:** Fetal eyes, Epigenetics, Histone acetylation, DNA methylation, Retinal development, Immunohistochemistry

## Abstract

**Purpose:**

The prenatal development of the human retina is a complex process orchestrated by several epigenetic mechanisms. This study investigated some global epigenetic markers (histone H3, histone H4, 5-methylcytosine, 5-hydroxymethylcytosine) during the fetal development of the human retina in 34 human eyes from 11 to 38 weeks of gestation.

**Methods:**

Immunohistochemical staining was performed to assess the distribution and intensity of the selected epigenetic markers across developmental stages. For the general retinal staining intensity, a score from 0 to 3 was used, whereas for the percentage of stained retinal nuclei, a score from 0 to 4 was applied. The two scores were multiplied resulting in the final staining score. The mean of the two graders was used for statistical analysis. Staining patterns were analyzed with regard to regional disparities (central versus periphery), distinctions among retinal cell layers, individual cell types, and changes over time.

**Results:**

The immunohistochemical staining reaction varied across developmental stages, showing regional disparities and distinctions among retinal cell layers and individual cell types. We observed consistent trends of decreasing staining scores for histone 3 and 4 acetylation and DNA methylation decrease with advancing gestational age. Significant differences among trimesters indicated dynamic epigenetic changes during fetal retinal development. Morphological analysis revealed distinct staining patterns that correlated with cellular morphology and differentiation levelsGganglion cells, amacrine cells, and Müller cells demonstratednotable similarities in staining patterns with regard to the developmental stage. Furthermore, differences between central and peripheral retina diminished with age.

**Conclusion:**

The study provides novel insights into the epigenetic regulation of retinal development in humans and underscores the importance of analyzing the epigenome at the cellular level for a comprehensive understanding. Understanding these epigenetic processes may pave the way for further investigations on retinal development and the communication between different cell types.

**Supplementary Information:**

The online version contains supplementary material available at 10.1007/s00417-025-07038-7.

## Abstract (highlights)

**Key messages:**

***What is known*:**

- Previous studies have demonstrated that the maturation of retinal cells is accompanied by dynamic changes in DNA methylation and histone modifications.

*****What is new***:**

- Global epigenetic markers vary significantly across retinal cell layers in fetal development.
- Histone 3 and 4 acetylation and DNA methylation decrease with advancing gestational age.
- Ganglion, amacrine, and Müller cells show notable similarities in staining pattern.

**Supplementary Information:**

The online version contains supplementary material available at 10.1007/s00417-025-07038-7.

## Introduction

### Retinal development in brief

The retina originates from the inner layer of the optic vesicle layer derived from the diencephalon (reviewed in [1]). The differentiation of retinal cells occurs in successive stages, involving multipotent progenitor cells, migration, and synaptogenesis [2]. The development of the retina progresses from central to peripheral and from inner to outer layers [3]. Amacrine cells differentiate first, followed by cone photoreceptors, horizontal, and ganglion cells, then rod photoreceptors, Müller, and bipolar cells [4].

### Epigenetics in brief

Epigenetic mechanisms, including DNA methylation, histone modifications, and non-coding RNAs, regulate gene expression and are potentially reversible, allowing dynamic changes under multifactorial influences [5]. These mechanisms are crucial during prenatal retinal development [6].

Histone modifications, particularly acetylation, influence gene expression by altering chromatin accessibility. Acetylation is more pronounced in undifferentiated retinal cells than in differentiated photoreceptor cells [7]. Other studies support this observation, indicating histone acetylation as a key factor in progenitor cell differentiation into photoreceptor cells [8–11]. DNA methylation also plays a pivotal role in retinal development, as animal models have shown that loss of DNA methyltransferases (DNMT) is associated with retinal abnormalities and dysfunction, emphasizing its importance for proper differentiation and retinal cell function [12–17]. Berdasco et al. found hypermethylation in promoter regions of transcription factors in adult retinal tissue [18]. During early development, transcription factors like PAX6 exhibited hypomethylation in their promoter regions, correlating with increased expression [18].

DNA hydroxymethylation, specifically the formation of 5-hydroxymethylcytosine (5hmC) from 5-methylcytosine (5mC), is another crucial epigenetic process. Studies in zebrafish suggest that disruption of TET enzymes, which catalyze hydroxymethylation, can lead to dysfunction in retinal cells [19]. Immunohistochemical studies in mouse retina further demonstrated dynamic and complementary distribution patterns of 5mC and 5hmC during development, with 5mC enriched in heterochromatic domains and 5hmC localized to euchromatic, transcriptionally active regions, underscoring their role in chromatin reorganization throughout retinogenesis [20].

### Aim of the study

This study aimed to investigate spatial and temporal epigenetic modifications in different retinal cell types of the fetal retina. To characterize global epigenetic changes, immunohistochemical staining for acetyl-histone H3, acetyl-histone H4, 5mC, and 5hmC was performed in 34 human fetal eyes at different developmental stages and compared to 11 adult retinal tissue samples.

## Materials and methods

### Material

**Fig. 1:**
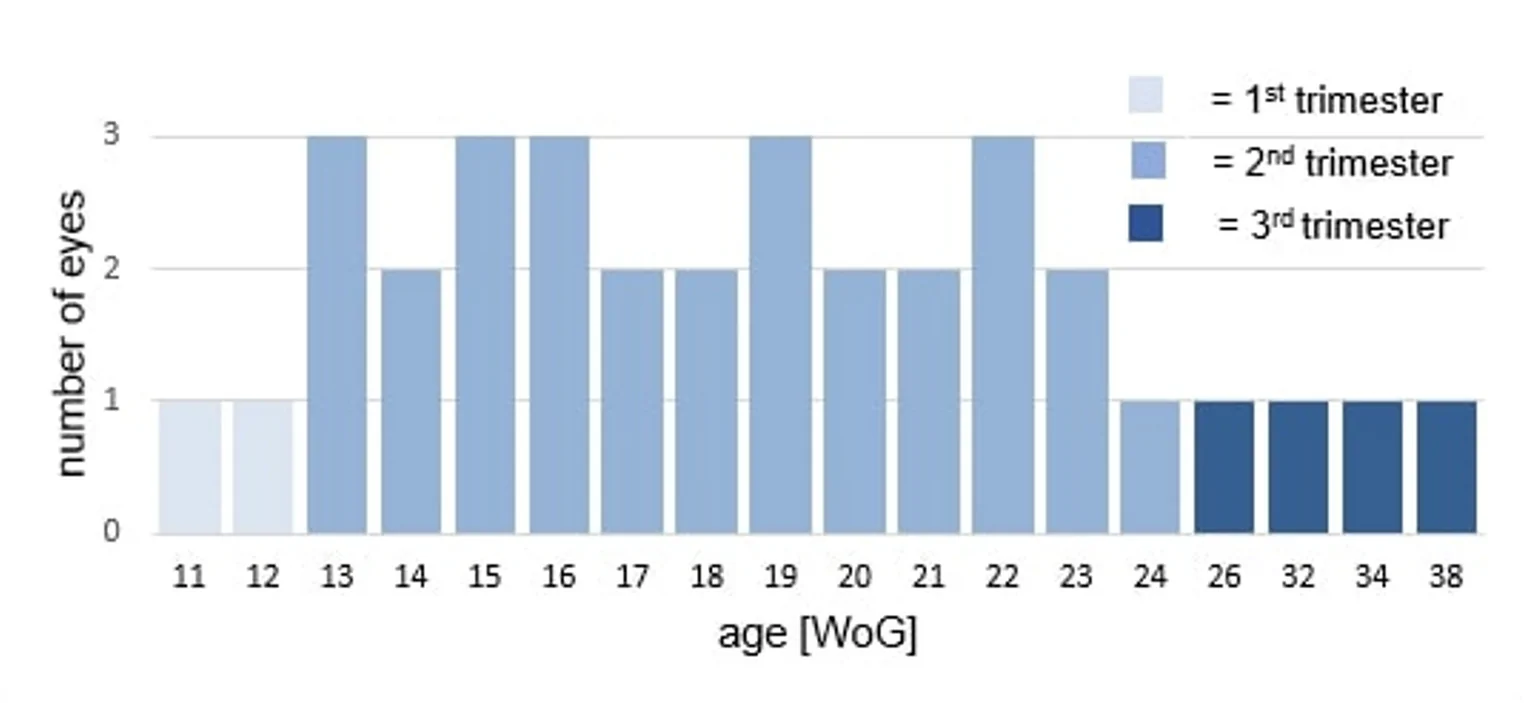
Overview of the number and age (in weeks of gestation, WoG) of the investigated fetal eyes

Formalin-fixed paraffin-embedded eyes from 34 human fetuses (age range: 11 to 38 weeks of gestation, WoG) were examined (Fig. 1). The cohort comprised two eyes from the first trimester, 28 eyes from the second trimester, and four eyes from the third trimester. Only one eye from each fetus was included into the study. The cohort consisted of 17 male and 17 female fetuses. Only eyes with minor retinal artifacts and morphologically normal retinal development were included. Eyes of fetuses with chromosomal abnormalities were excluded, as these may lead to epigenetic alterations and may differ from the normal population. The cause of death was fetocide in 21 cases, postnatal in 7 cases, intrauterine in 5 cases, and unknown in one case. The fetal eyes were obtained for diagnostic purposes during the pediatric pathologic autopsy.

Eleven adult eyes (mean age: 67 years, male: *n* = 7, female: *n* = 4) with morphologically normal retina were used as controls for comparison. The eyes were enucleated for medical reasons such as therapy-resistant corneal ulceration with severe inflammation of the anterior segment.

Written informed consent was obtained from the parents resp. adult patients in all cases. The research was conducted in accordance with the tenets of the Declaration of Helsinki (ethics approval of the University of Bonn, number: 227/11).

### Methods

The formalin-fixed paraffin-embedded eyes were routinely stained for H&E (hematoxylin and eosin; Fa. Merck, Darmstadt, Germany) and PAS (periodic-acid Schiff reaction; Fa. Merck, Darmstadt, Germany). Immunohistochemical staining was performed for anti-acetyl-Histone H3 (anti-H3Ac; polyclonal rabbit (IgG) anti-human; Cat.-No: 06–599; Merck Millipore, Darmstadt, Germany; dilution 1:200), anti-acetyl-Histone H4 (anti-H4Ac; polyclonal rabbit (IgG) anti-human; Cat.-No: 06–866; Merck Millipore, Darmstadt, Germany; dilution 1:2500), anti-5-methylcytosine (anti-5MeC; monoclonal mouse (IgG1) all species; clone 4111; Cat.-No: NBP2-42814; Novus Biologicals, Darmstadt, Germany; dilution 1:150) and anti-5-hydroxymethylcytosine (anti-5hMeC; monoclonal mouse (IgG2b) all species; clone 317; Cat.-No: MA5-23525; Thermo Fisher Scientific, Dreieich, Germany; dilution 1:1500). 5 μm thick sections were deparaffinized and rehydrated. Heat-mediated antigen retrieval was performed in a microwave (750 W) with 0.01 citrate buffer (pH 6.0) for 3 × 10 min. Washing steps were performed with Tris buffer (pH 7.6). Samples were incubated with the primary antibody overnight at 4 °C. Endogenous peroxidase was blocked with 3% hydrogen peroxide for 10 min, followed by incubation with a secondary antibody. In the case of anti-5MeC, a biotinylated secondary antibody was used (anti-Mouse/Rabbit IgG, biotinylated, Art.-Nr.: ZU114; Nordic MUbio, Susteren, the Netherlands, dilution 1:50). A polymer-conjugated secondary antibody was used for anti-H3Ac, anti-H4Ac and anti-5hMeC (Mouse and Rabbit Specific HRP/AEC IHC Detection Kit – Micropolymer (ab236467); Abcam, Cambridge, United Kingdom). Both secondary antibodies were used in combination with horseradish peroxidase (HRP) and 3-amino-9-ethylcarbazole (AEC) as chromogen. Counterstaining was performed with Mayer’s hemalaun (Carl Roth, Karlsruhe, Germany).

### Analysis

**Fig. 2:**
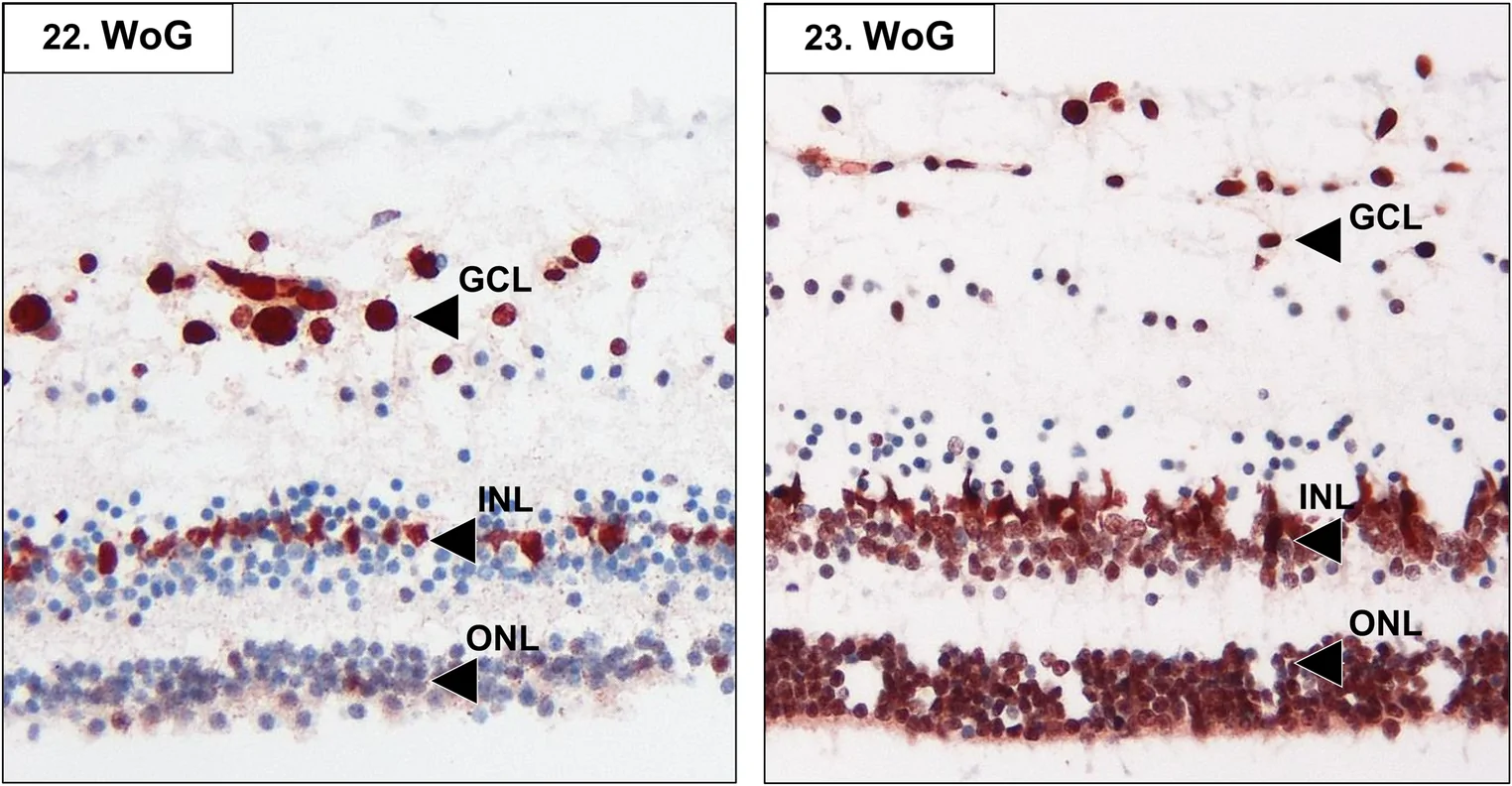
Representative immunohistochemical stainings illustrating the immunohistochemical scoring system. In the GCL at 22 WoG, more than 50% but less than 75% of nuclei (Score 3) showed strong staining intensity (Score 3), resulting in a staining score of 9. In the INL at 22 WoG, clearly less than 25% of nuclei (Score 1) displayed strong staining intensity (Score 3), corresponding to a staining score of 3. In the ONL of another specimen at 23 WoG, more than 75% of nuclei (Score 4) exhibited strong staining intensity (Score 3), yielding the maximum staining score of 12

The samples were analyzed for the immunohistochemical staining reaction of retinal cells in the central vs. the peripheral retina. The intensity of the immunohistochemical staining reaction was assessed by two independent examiners (MTJ, MCHC) using semiquantitative scores for the staining intensity and the percentage of labelled cells. Staining intensity was scored from 0 to 3 (0: no staining reaction, 1: weak staining reaction; 2: moderate staining reaction; 3: strong staining reaction) and the percentage of stained retinal nuclei was scored from 0 to 4 (0: no staining reaction, 1: staining reaction in less than 25% of nuclei, 2: staining reaction in 25% to 50% of nuclei, 3: staining reaction in 50% to 75% of nuclei, 4: staining reaction in more than 75% of nuclei) (Fig. 2). The final staining score was the product of these two scores, and the mean score was used for statistical analysis (Table 1). A deviation of more than two points in the color score was observed in less than 10% of cases. Cell type patterns were documented by localization and morphology.

**Table 1:** Presentation of the statistical values of the immunohistochemistry score (mean, standard deviation [SD], and median) for Anti-H3Ac, Anti-H4Ac, Anti-5MeC, and Anti-5hMeC in different retinal regions (central retina, peripheral retina), and developmental stages (1st, 2nd, and 3rd trimester, as well as adult). Fetal eyes were analyzed separately for central and peripheral retina, whereas adult eyes were not subdivided

Antibody	Retinal Area	Age Group	Mean	SD	Median
Anti-H3Ac	Central Retina	1 st trimester	5	1	5
		2nd trimester	9.41	2.40	10
		3rd trimester	6.92	2.92	5
	Peripheral Retina	1 st trimester	4.75	0.96	4.5
		2nd trimester	8.49	2.44	9
		3rd trimester	6.67	2.36	7
	Unspecified	Adult	11.09	1.59	12
Anti-H4Ac	Central Retina	1 st trimester	4.33	0.58	4
		2nd trimester	7.13	3.31	7.5
		3rd trimester	5.5	2.95	6.25
	Periphiral Retina	1 st trimester	4.63	2.14	4
		2nd trimester	6.59	3.35	6
		3rd trimester	5.92	2.95	6.25
	Unspecified	Adult	11.91	0.52	12
Anti-5MeC	Central Retina	1 st trimester	1.5	0.5	1.5
		2nd trimester	8.91	2.6	9
		3rd trimester	5.71	1.68	5
	Peripical Retina	1 st trimester	3.63	2.75	3.75
		2nd trimester	8.38	2.67	9
		3r trimester	7.21	3.09	9
	Unspecified	Adult	11.55	12	1.09
Anti-5hMeC	Central Retina	1 st trimester	3	0.5	3
		2nd trimester	6.28	2.98	6
		3rd trimester	3.5	1.34	3.5
	Peripheral Retina	1 st trimester	3.5	1..29	3.5
		2nd trimester	6.07	2.89	6
		3rd trimester	3.79	2.07	4

Statistical analysis was performed using IBM SPSS 27 with Pearson correlation and Mann-Whitney U-test. The cohort was divided into three age groups: 1st trimester (1–12 WoG), 2nd trimester (13–24 WoG), and 3rd trimester (24–38 WoG), with *p* < 0.05 considered significant. Pearson correlation was used to examine trends across the full age range of fetal eyes, capturing continuous relationships between age and immunohistochemistry scores. The Mann-Whitney U test was applied to compare independent groups, specifically the second and third trimester, to evaluate differences between these developmental stages.

## Results

**Fig. 3:**
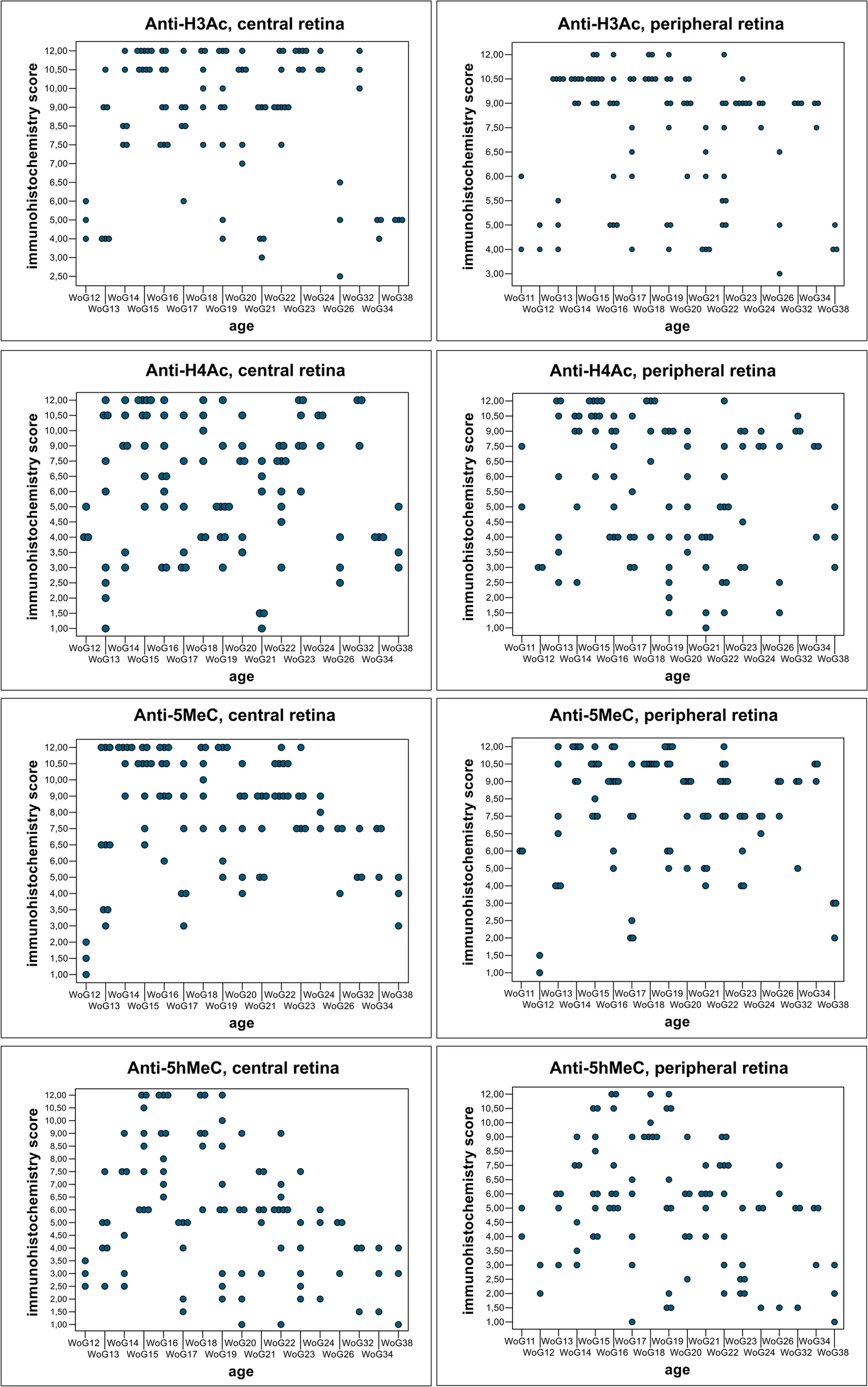
Dot plot representation of the immunohistochemistry score for the antibodies Anti-H3Ac, Anti-H4Ac, Anti-5MeC, and Anti-5hMeC across individual gestational weeks. Each dot corresponds to the immunohistochemistry score of a single sample, illustrating the distribution and variation of antibody staining intensities throughout gestational development. WoG: week of gestation

A staining reaction was observed in all samples examined, showing variation across developmental stages, retinal layers, and cell types (Fig. 3).

### Age-related retinal development

**Fig. 4:**
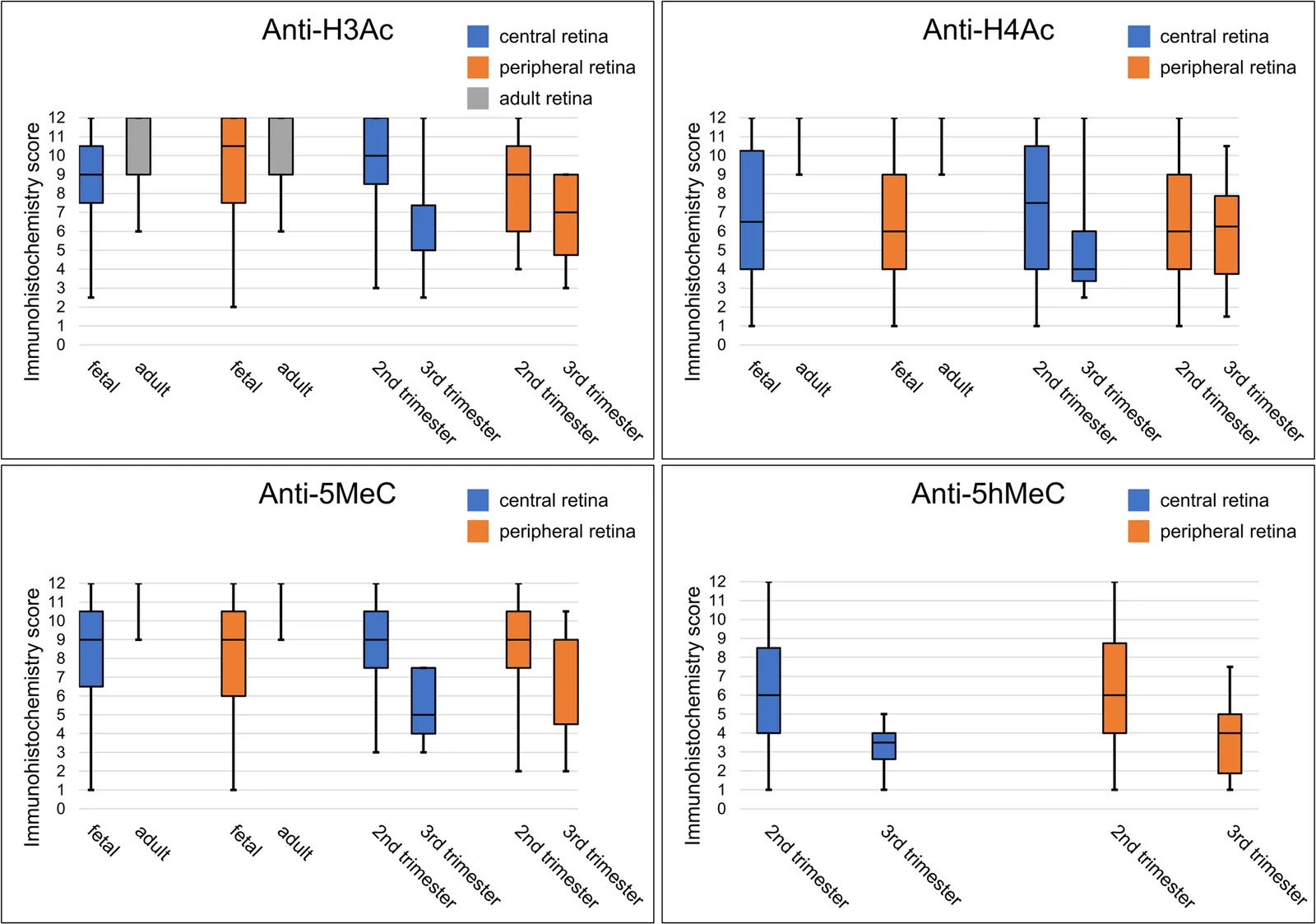
Comparison with boxplots of the immunohistochemistry score in fetal and adult retina. Fetal retina is presented separately for central and peripheral regions, while adult retina is shown without such subdivision, as no regional differences between central and peripheral areas were observed. Comparisons with adult retina were performed only for antibodies against H3Ac, H4Ac, and 5MeC; no staining with 5hMeC was carried out in adult samples. Fetal retina is further compared between the second and third trimester, each subdivided into central and peripheral regions. The data indicate stronger staining in the second trimester compared to the third, and consistently high staining intensity in adult retina

Staining intensity decreased with increasing gestational age across all antibodies and retinal layers (outer nuclear layer (ONL), inner nuclear layer (INL), ganglion cell layer (GCL), *p* < 0.01). Statistical analysis was only performed between the second and third trimesters, as the number of first-trimester samples was very limited (*n* = 1–2) (Fig. 4).

### Central retina

Significant differences were observed for anti-H3Ac, anti-H4Ac and anti-5hMeC. Eyes from the second trimester had significantly higher color scores compared to eyes from the third trimester (*p* = 0.006 for anti-H3Ac, *p* = 0.001 for anti-5MeC, *p* = 0.002 for anti-5hMeC). A similar trend was observed for anti-H4Ac, but no significant differences were observed (*p* = 0.120).

Further analysis of individual retinal layers revealed a significant difference for anti-5MeC in the ONL with eyes from the second trimester showing significantly higher color scores with anti-5MeC than eyes from the third trimester (*p* = 0.023).

### Peripheral retina

Significant differences were found for anti-H3Ac and anti-5hMeC. For these antibodies, eyes from the second trimester showed a stronger staining reaction than eyes from the third trimester (*p* = 0.012 for anti-H3Ac and *p* = 0.031 for anti-5hMeC).

Separate examination of individual retinal layers indicated a significant difference between age groups for anti-5hMeC in the ONL with eyes from the second trimester showing significantly higher color scores than eyes from the third trimester (*p* = 0.033).

### Spatial distribution

A strong positive correlation between staining intensities in central and peripheral retina was observed. This correlation refers to the similarity in staining intensities achieved by the antibodies in the respective cell layers of the central and peripheral retina. When considering all antibodies and retinal cell layers together, the correlation was *r* = 0.793 (*p* < 0.01), indicating a high degree of similarity between the staining patterns in the central and peripheral regions.

When analyzing individual retinal layers separately, the ONL exhibited the highest correlation, followed by the INL (*r* = 0.793) and the GCL (*r* = 0.770). These correlations were statistically significant in all cases (*p* < 0.01).

When considering individual antibodies, a positive correlation was observed between the central and peripheral retina in each case. The strongest correlation was found for anti-5MeC in the GCL (*r* = 0.914), whereas the weakest correlation was found for anti-H4Ac in the GCL (*r* = 0.545). Significance was reached in all cases (*p* < 0.01).

### Adult eyes

Eleven adult eyes were used to compare the staining of anti-H3Ac, anti-H4Ac and anti-5MeC antibodies between fetal and adult retinal tissue. In the adult specimens, no differences in staining intensity were observed between central and peripheral retina nor between different cell types.

### Morphological evaluation

The staining intensity was not solely dependent on the general retinal staining reaction; distinct patterns were also observed within layers and between different cell types. The identification of retinal cell types was largely based on the findings of Ida Mann, who described the layering and morphology of retinal cells during fetal development at the beginning of the 20th century [3].

**Fig. 5:**
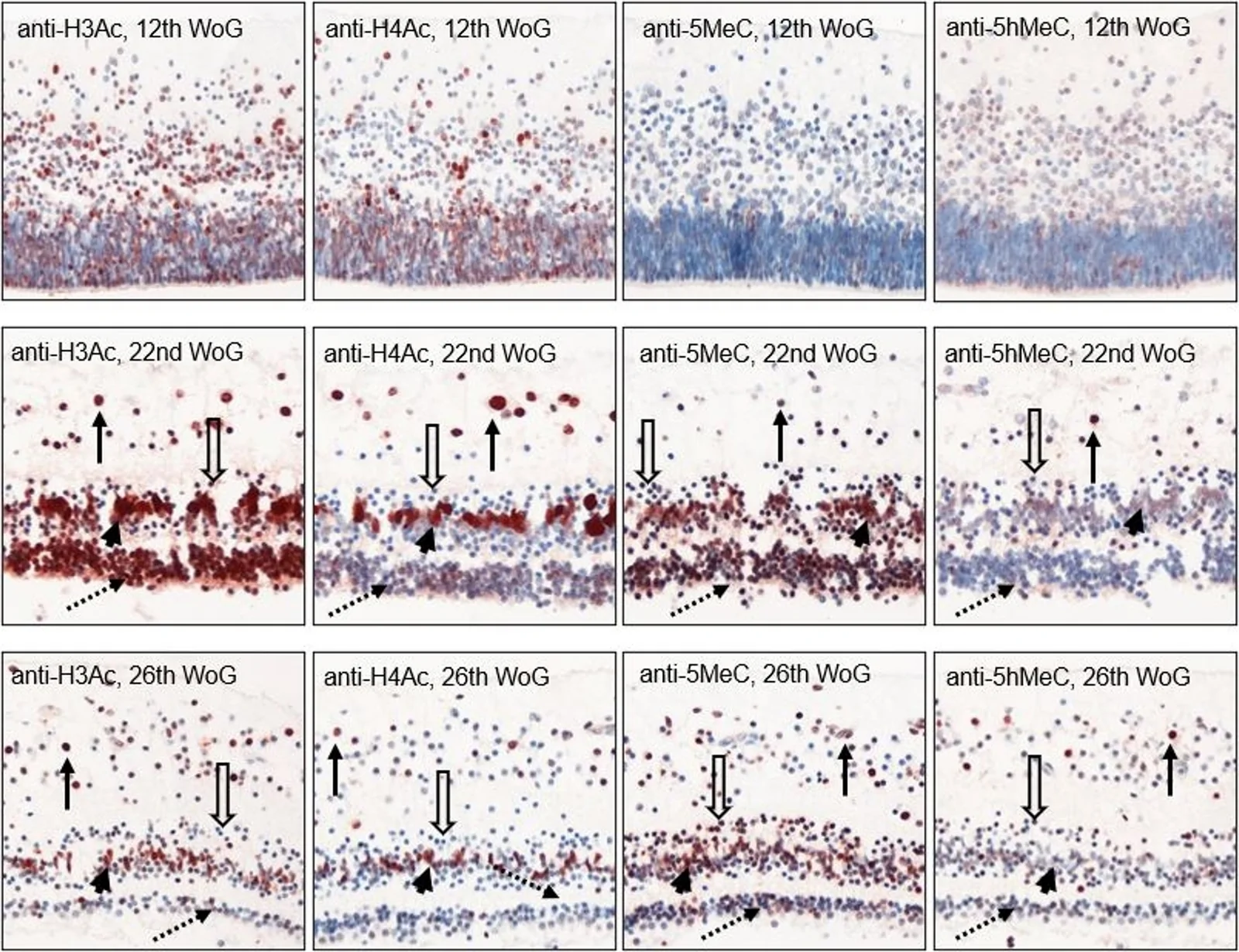
Staining reaction in the central retina at different developmental stages. This figure illustrates the retina of fetal eyes at the 12th, 22nd, and 26th WoG. Over time, the organization of retinal layers becomes increasingly defined. At 12 weeks, the retina is characterized by the presence of inner and outer neuroblastic layers, without clear differentiation of individual cell types. By 22 and 26 weeks, the maturation of the retina allows for the identification of distinct cell types, including ganglion cells (arrow), amacrine cells (blank arrow), Müller cells (arrow head) and photoreceptor cells (dotted arrow). Magnification: 100x

In early developmental stages, a characteristic pattern emerged: cells with round-shaped nuclei (indicating higher differentiation) showed more intense staining compared to cells with elongated or oval nuclei (indicating lower differentiation). At 12 WoG, the retina is characterized by the presence of inner and outer neuroblastic layers, without clear differentiation of individual cell types with the round-shaped nuclei of the inner and less frequent outer neuroblastic layer being more intensely stained than the oval-shaped, densely packed nuclei of the ourter nuclear layer (Fig. 5). This difference highlights the correlation between cell morphology and degree of differentiation. Over time, the organization of retinal layers becomes increasingly defined.

**Fig. 6:**
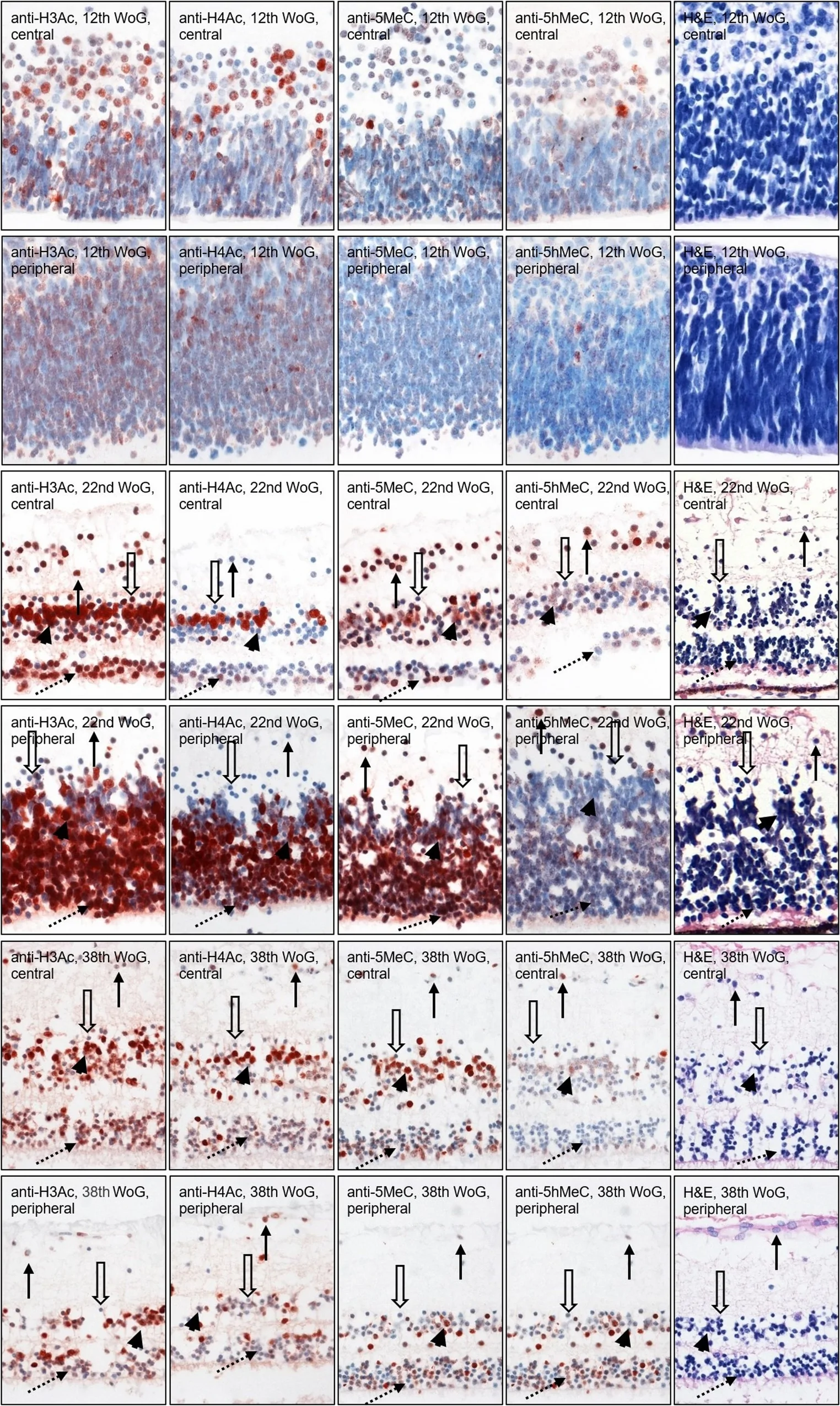
Staining reaction of the retinal layers in the central retina compared to the peripheral retina at different developmental stages, compared to H&E staining. The image depicts the retina of fetal eyes at 12, 22, and 38 weeks of gestation (WoG). Central retinal regions develop earlier than peripheral regions, leading to more advanced cellular differentiation in central sections. During early developmental stages, cells with round nuclei, indicative of more advanced differentiation, show stronger staining compared to cells with oval nuclei. This is particularly evident in the central retina stained with anti-H3Ac and anti-H4Ac. Significant differences in staining intensity between central and peripheral retina can be observed within the same specimen. For instance, at 22 weeks of gestation, anti-H4Ac staining reveals that photoreceptor cells (dotted arrow) in the central retina are only faintly stained, while those in the periphery are strongly stained. Arrow: ganglion cells; blank arrow: amacrine cells; arrow head: Müller cells. Magnification 100x

By 22 and 26 weeks, the maturation of the retina allows for the identification of distinct cell types, including ganglion cells, amacrine cells, Müller cells and photoreceptor cells. In particular, ganglion cells and amacrine cells often had a similar level of color intensity and tend to show contrasting characteristics compared to other cell types in the same context (Fig. 6), where the strong staining of amacrine cells together with ganglion cells at week 22 was particularly evident in the central retina stained with anti-5MeC.

Furthermore, an intriguing parallel arose between Müller cells and amacrine cells as well as ganglion cells. This similar staining between ganglion cells and Müller cells was particularly evident at 22 WoG (Fig. 5) when stained with anti-H4. In the case of photoreceptor cells, a heterogeneous staining pattern was observed, setting them apart from other retinal cell types: in some specimens, these cells displayed intense staining, whereas in others they showed weaker staining.

Another intriguing observation was the variability in staining intensity between different cell types. In some cases, a particular cell type displayed strikingly strong staining, while adjacent cells were only weakly stained. These differences may provide insights into the specialized functions of individual cell types within the retinal structure.

Differences between central and peripheral retina were also apparent: staining patterns that initially appeared in the central retina (which is typically more advanced in development) later emerged in the peripheral retina as it continued to mature. This phenomenon was particularly evident in older eyes and highlights the complexity of retinal development and differentiation over time.

## Discussion

This study investigated the links between epigenetics and fetal retinal development in human fetal FFPE tissue. Previous research on this topic has often focused on the whole retina without considering the retinal cell layers or cell types. In addition, model organisms such as mice are often used which is somehow justified due to the conservation of the epigenome between mice and humans; however, direct applicability to human retinal development is limited [6].

A key contribution of the present study is that it was performed on the whole retina of human fetal eyes. To the best of our knowledge, this study is the first to thoroughly explore epigenetic factors in human fetal eyes throughout the entire fetal development using the technique of immunohistochemistry. This approach allowed differentiation between various retinal cell types and developmental stages, unlike previous studies that often examined whole retinas without such distinctions.

Recent studies have demonstrated that individual retinal cell types have distinct epigenetic profiles [7, 21–26]. Our results are consistent with these findings, revealing cell-type dependentexpression patterns of the investigated epigenetic global markers across different cell types as fetal development progresses.

Furthermore, the use of single-cell techniques has highlighted variability in the epigenome even among individual cells within the same retinal cell layer suggesting that epigenetic mechanisms are not only cell-type-specific but may also vary at the single-cell level [27–29]. Our study confirms this variability by the co-staining of different cell types during the ocular development.

Ganglion cells and amacrine cells displayed similar staining patterns across all antibodies used, indicating shared global epigenomic modifications and a close developmental association. These cell types often demonstrated contrasting staining behavior compared to others, supporting previous findings that suggest a modulatory relationship during development [30–32]. Müller cells also showed similarities to ganglion and amacrine cells, albeit to a lesser extent. The developmental link between Müller and amacrine cells has been noted in the murine retina [33], with Müller cells modulating spontaneous electrical impulses during retinal development [34].

Our results demonstrate that early developmental stages are characterized by higher histone acetylation, DNA methylation, and hydroxymethylation, emphasizing the dynamic nature of epigenetic regulation during retinal development. With regard to histone acetylation, this has been confirmed in previous studies [7]. However, our results partially contradict previous studies, as no decrease in DNA methylation during the fetal period has been demonstrated to date [6, 35, 36]. Furthermore, the observed correlation between global 5MeC and 5hMeC expression differs from previous findings, highlighting the need for cautious interpretation due to differences in tissue types and developmental stages [37].

The study also underscores that epigenetic patterns depend not only on cellular differentiation but also on spatial distribution in the retina. Differences in epigenetic markers between the central and peripheral retina were observed during fetal development, but these differences diminished with increasing age.

The positive correlation in color scores among all antibody pairs suggests a consistent global behaviour of the epigenetic mechanisms studied, in particular a high correlation in H3 and H4 histone acetylation. Despite the generally opposing effects of DNA methylation and histone acetylation on gene expression, some studies reveal a paradoxical positive relationship [38–41]. While previous research has demonstrated associations between DNA hypermethylation and histone hypoacetylation, the interactions between DNA methylation, histone acetylation, and gene expression are complex and do not strictly follow established patterns [7, 40, 42]. In particular, hypermethylation in intragenic regions outside of gene promoters can positively correlate with active transcription when associated histone acetylation is taken into account [42].

## Conclusion

Our study investigated the role of epigenetics in human fetal retinal development. We observed dynamic changes in the immunohistochemical staining pattern of global epigenetic markers during retinal development. A co-immunostaining of different retinal cell types indicated shared epigenetic modifications and developmental connections. Our results also highlight the importance of spatial distribution within the retina, revealing differences between central and peripheral regions during fetal development. In conclusion, our study provides valuable insights into the epigenetic regulation of human retinal development and deepens our understanding of the molecular mechanisms underlying ocular tissue morphogenesis.

## Supplementary Information

Below is the link to the electronic supplementary material.

Supplementary Material 1 (DOCX.1.81 MB)
